# Seeking safety: a focus group study of young adults’ cannabis-related attitudes, and behavior in a state with legalized recreational cannabis

**DOI:** 10.1186/s12954-020-00442-8

**Published:** 2020-11-26

**Authors:** Nada Amroussia, Mika Watanabe, Jennifer L. Pearson

**Affiliations:** 1 Faculty of Health and Society, Malmö University, Malmö, Sweden; 2 Division of Social and Behavioral Health, University of Nevada, Reno, USA; 3 Division of Health Administration and Policy, University of Nevada, Reno, USA; 4 Division of Social and Behavioral Health/Health Administration and Policy, University of Nevada, Reno, USA; 5 Department of Health, Johns Hopkins Bloomberg School of Public Health, Behavior, and Society, Baltimore, MD USA

**Keywords:** Recreational cannabis legalization, Cannabis policy, Cannabis, Young adult, Nevada

## Abstract

**Background:**

Only July 1, 2017, Nevada became the fifth US state to allow the legal sale of recreational cannabis products for adults ages of 21 and over. This study investigates young adults’ cannabis-related attitudes, perceptions, and behaviors in a state where recreational and medical cannabis use was recently legalized.

**Methods:**

We conducted 8 focus groups stratified by cannabis use (regular users, occasional users, and nonusers) with 32 college students ages 18 to 24. Data were analyzed using the inductive qualitative thematic analysis method.

**Results:**

Four themes emerged during analyses: “sort of legal,” “mitigating harm through legalization,” “Increasing acceptance,” and “seeking safety when purchasing cannabis.” Despite their limited knowledge of cannabis regulation, the majority of the participants supported recreational cannabis legalization from a harm reduction perspective. Most participants did not believe that cannabis legalization had affected their use behavior. However, participants, especially cannabis users, perceived that recreational cannabis legalization created a context where cannabis use was legally, socially, and behaviorally “safer” than in an illegal context, even for those below the legal age of sale.

**Conclusions:**

Most studies focus on the role of perceived health risk on cannabis use. If there are population-level long-term effects of recreational cannabis legalization on use behavior, findings suggest that they will be mediated by the perceived legal, social, and behavioral risk of using cannabis.

## Background

Recent legalization of recreational cannabis sales in several US states has fueled discussion about the effect of legalization on young adults' cannabis use attitudes and behaviors [1]. Young adults have the highest prevalence of ever (51.8%) and past 30-day cannabis use (33.0%) of any age group, and cannabis is the second most commonly used substance after alcohol among young adults [2]. While some adults use cannabis without harm [3], long-term and heavy cannabis use (defined as every day or almost every day use) [4] is associated with dependence, altered brain development, and diminished life satisfaction [5]. Given the developmental importance of young adulthood in shaping individuals’ future health behavior, understanding young adults’ behavioral response to cannabis legalization is of utmost public health interest.

Despite the importance of this question, quantitative research on the relationship between cannabis legalization and adult cannabis use has yielded mixed results, and few studies have reported results for young adults [6]. While some studies found an increase in lifetime use [6], past-30 day use [6, 7], and frequency of use [7] after the legalization of medical or recreational cannabis [6, 7], other studies found no significant effect on adult behavior [8–10]. These disparate results might be explained by different definitions of the cannabis use outcome (e.g., changes in past 30-day use, lifetime use, etc.), conflation of medical and recreational laws, higher than average cannabis use prevalence preceding legalization in a state, or differences in policy implementation. Additionally, most of these studies were conducted within a relatively short period after change in cannabis laws; change in cannabis regulations might have long-term rather than short-term effects on adults’ behavior [11].

Previous studies on the effects of cannabis legalization specifically on young adults' cannabis attitudes and use have been predominately quantitative and often focus on changes in perceived harm (i.e., the perception of harm or damage to the physical body associated with using cannabis) or perceived availability [6–10, 12]. Beyond perceived harm, recreational legalization might also stimulate changes in perceived social or legal risk of use [6]. *Perceived social risk* is defined as an individuals’ belief as to how others will react to their cannabis use, while *perceived legal risk* is defined as an individual’s belief as to the severity of legal punishment associated with their cannabis use [13].Assessing these additional constructs will explain why some young adults use cannabis, or why some of legal purchase age choose the black market over the legal dispensary.

Understanding the effect of recreational cannabis legalization on young adults' cannabis-related perceptions, attitudes, and behaviors will contribute to our understanding of how legalization affects cannabis use prevalence and ultimately the public health consequences of legalization [14]. The overall aim of this study was to understand young adults’ cannabis-related attitudes, perceptions, and behaviors in Nevada. Like many states [15], Nevada legalized cannabis use for patients suffering from serious health problems in the early 2000s, expanding medical cannabis use to patients with additional aliments in 2013 [16]. In November 2016, Nevada passed the *Regulation and Taxation of Marijuana Act,* which legalized the purchase, possession, and consumption of recreational cannabis for adults ages 21 and over. Legal recreational cannabis sales began on July 1, 2017 [17], and as of 2018, Nevada had over 60 licensed recreational cannabis dispensaries [18]. Given this legal context, our specific aims were: (1) to explore young adults’ understanding of Nevada’s recent changes to state cannabis law; and (2) to elicit young adults’ reflections on how recreational cannabis legalization has affected how they perceive, purchase, and use cannabis.

## Methods

### Conceptual framework—a harm reduction perspective to understanding young adult cannabis use

The 2016 Nevada statute legalizing recreational cannabis sales recognized legalization as a means to reduce incarceration and remove cannabis “from the domain of criminals” so that it may “be regulated under a controlled system” [19].We used a similar harm reduction perspective in the study’s conceptualization, design, and analyses to understand the individual- and societal-level benefits and harms young adults perceive as a consequence of legal recreational cannabis sales.

### Study design

The purpose of focus group research is to elicit common knowledge and capture different views and perspectives within a specific population or subgroup [20], using group interactions to collect data that might not be available using other qualitative methods. In this study, we used focus groups to gain insight into young adult college students’ knowledge and perceptions of cannabis laws, as well as their understanding of how Nevada’s cannabis laws affect their attitudes about and use of cannabis.

### Study setting

The study was conducted at the University of Nevada, Reno (UNR), a public university. Reno is located in northern Nevada with an estimated population of 248,853 in 2017 and a median per capital income of $48,815 in 2016 [21]. The total number of UNR students enrolled in fall 2018 was 19,911, with 54.2% women students and 82.9% undergraduate students. While the majority of UNR students are from Nevada (70.5%), a significant proportion also come from other states [22].

### Eligibility and recruitment

We recruited a purposively selected sample of college students using flyers posted around campus, which directed students to an online screening questionnaire on Qualtrics. Participants were eligible if they were (1) between the ages of 18 and 24, and (2) undergraduate or graduate students. Eligible individuals were invited to participate in the study and asked to provide their emails for scheduling purposes. Participants were divided into three groups based on self-reported patterns of cannabis use: regular users, occasional users, and non-users. These cannabis use categories were based on participants’ responses to the screener question, “Do you currently use any marijuana product?” Response options included “yes, regularly” (regular users), “yes, occasionally” (occasional users), and “no, not at all” (non-users).

### Procedure

We conducted 8 focus group discussions between December 1st, 2017, and March 8th, 2018: 2 non-user groups, 3 occasional user groups, and 3 regular user groups. Participants provided written informed consent prior to data collection. All focus groups were conducted in a private meeting room to protect participants’ privacy. Each participant provided a pseudonym for use during the focus group and data analyses. The results presented in this paper include the participants’ pseudonyms.

Before the focus group discussion began, participants completed pre-focus group questionnaires with items on socio-demographic characteristics, history of cannabis use, and perceived parental history of cannabis use. We used a facilitator guide with open-ended questions grouped in two sections for users and non-users. All groups were attended by a facilitator and 1–2 co-facilitators. The mean duration of the focus groups was 50 min. Facilitators and co-facilitators took notes and made memos during the groups. The focus groups were conducted in English and were audio-taped. Data collection was continued until saturation was achieved, i.e., no new information has emerged during the focus group. Each participant received a $20 Starbucks gift card after completing the focus group. The study was approved by the Institutional Review Board of the University of Nevada, Reno.

### Data analyses

Focus groups were transcribed verbatim by the first author. We analyzed the transcripts using an inductive thematic analysis approach following Braun and Clarke [23]. First, the first author read the transcripts several times to gain familiarity with the data. Then, the transcripts were coded line by line. The second author reviewed the codes and both authors examined the emergent codes to look for thematic patterns in the data. Five thematic patterns related to the study aims emerged during this process, which were later refined into 4 themes using the harm reduction framework. We completed analyses using Opencode 4.03 [24]. Quote selection was based on two criteria: the clarity of the quotes, and their ability to capture the essence of the focus groups discussion, i.e., a quote reflected participants’ overall perceptions and attitudes.

## Results

### Study population

Thirty-two participants agreed to participate in the study: 12 non-users, 10 occasional users, and 10 regular users. More than half of the participants were women (53.1%), and more than two-thirds were aged between 18 and 20 years old (68.7%). Nearly half (43.8%) of the participants were non-Latino/a white. Most of the participants were undergraduates (93.8%) and US-born (90.6%). Overall, 25% of the participants believed that their parents used cannabis in the past and 18.8% of the participants reported that their parents are current cannabis users (Table 1).

**Table 1:** Summary of participants’ characteristics

Participants’ characteristics	% (total = 100%)	N (Total = 32)
Gender		
Women	53.1	17
Men	46.9	15
Age		
Between 18 and 20	68.7	22
21 or over	31.3	10
Race/ethnicity		
White non-Latino/a	43.8	14
Black non-Latino/a	12.5	04
Latino/a	21.9	07
Other races/ethnicities	21.9	07
Born in the USA		
Yes	90.6	29
No	09.4	03
Studies		
Undergraduate student	93.8	30
Graduate student	06.2	02
Use pattern		
Regular user	31.2	10
Occasional user	31.2	10
Non-user	37.5	12
Parent cannabis use—past		
Yes, at least one parent	25.0	08
No, both parents	59.4	19
I don’t know	15.6	05
Parent cannabis use—current		
Yes, at least one parent	18.7	06
No, both parents	71.9	23
I don’t know	09.4	03

Four themes emerged during the data analysis: “sort of legal,” “mitigating harm through legalization,” “increasing” and “seeking safety *when purchasing cannabis*.”

### Sort of legal

In this theme, participants discussed their understanding of the boundaries of legal recreational cannabis use in the state. Participants were aware of changes in Nevada’s cannabis laws, but the depth of their knowledge varied significantly, and the majority of participants had limited knowledge of regulations. For example, participants were not familiar with the legal limit for individual cannabis possession. If participants were familiar with specific aspects of recreational cannabis legalization, they were only vaguely aware of the details of the law.

Not tons…I mean…I know that like recreationally you can have it over the age of 21…and… there's a certain amount that you are able to have. I don't know the number…

(Olivia, regular user, focus group discussion [FGD] 5)

Participants perceived recreational cannabis use as "sort of legal" (*Monica, occasional user, FGD1*) due to the conflict between state and federal law, the restricted legal age of use (21 +), and limitations on where one could legally use cannabis (i.e., private homes only).

Some participants referred to how other legal substances such as alcohol and tobacco are regulated as a way to understand how cannabis is regulated.

Then, you can consume it [cannabis] in private but not in public…like with alcohol…you're not able to do in the street.

(Robert, occasional user, FGD1)

So, you can't smoke and drive cause they're going treat it like DUI [driving under the influence] like alcohol.

(Dom, occasional user, FGD 4)

### Mitigating harm through legalization

This theme illustrates how participants discussed and viewed legalization from a harm reduction perspective. Regular and occasional cannabis users had positive perceptions of legalization. They perceived legal recreational cannabis as a source of beneficial tax revenue because “*it gives a lot of good things for our community*" (*Ben, regular user, FGD 8*). This perception was also shared by nonusers who reported taxation as a positive aspect of recreational cannabis legalization. Some users also perceived recreational cannabis legalization as a step toward decreasing use of other drugs and encouraging less risky cannabis use. Participants understood “less risky” cannabis use as avoiding the legal consequences of cannabis use, as well as having better control over their own use and using the products responsibly.

I am supporter of the legalization because I think it's very similar to…alcohol in that…it's safe when used responsibly… I grew up in Virginia where it was really super illegal and…it was like a felony… the consequences for getting caught with marijuana were really… really great. I think that it was kind of disproportionate consequence for the action. So, I am definitely supportive of the legalization.

(Sally, occasional user, FGD 7)

While occasional and regular users widely approved of recreational cannabis legalization, nonusers had ambivalent attitudes toward legalization. The majority of nonusers considered legal cannabis use as “safer” than illegal use and related recreational cannabis legalization to avoiding drug dealers and thus exposure to other illegal substances in the black market. Nonusers also perceived post-legalization cannabis use was “safer” because the risk of arrest had decreased, leading to fewer negative repercussions for users.

My viewpoint on it being recreational is a little bit safer than when it was illegal…just because the main argument against cannabis is…it was a gateway. (…) if their [cannabis users’] dealers didn't have weed, they offer them something stronger and… That’s how you get the gateway thing because of lack of availability of legal cannabis.

(Ingrid, nonuser, FGD2)

Nevertheless, a few of nonusers disapproved of legalization and perceived strict cannabis regulations as effective measures to reduce use.

I mean…just because it [cannabis] is a plant…it doesn't mean it's good. Individuals can be stupid. So, sometimes the government has to step in for the best of the population and say, "No don't do this!.

(James, nonuser, FGD6)

### Increasing acceptance

In this theme, participants discussed how legalization contributed to normalization of cannabis use. Participants agreed that recreational cannabis legalization has loosened community norms concerning cannabis use, especially among young people. Participants also described less negative social pressure concerning cannabis use from their families post-legalization, reflected in increased acceptability of cannabis use.

Definitely with family…like when we get in a family gathering or something like that…it's much more a topic of conversations…kinda like a joke…(…) It's not a super taboo thing either.

(Ben, regular user, FGD 8)

Some participants perceived that cannabis products were more accessible and available post-legalization, especially edible cannabis products.

I think I consume more [cannabis] now that it's available because I had really stopped smoking completely before that. But, I think a lot of it, it has to do with just I don't enjoy smoking…and now edibles are available I guess.

(Sally, occasional user, FGD 7)

While participants related legalization to increased availability of cannabis products and social acceptance of cannabis use, most participants did not believe that cannabis legalization has triggered change in their use behavior. Some regular and occasional users mentioned that they noticed an increase in their cannabis use after legalization, whereas others did not believe that their use was affected by the change.

I mean, I've only been like recreationally using it the past two years. So, I am not really…I am not super experiencing it…But, I don't think I changed the way I used it or how accessible… I think it is pretty much the same.

(Imani, occasional user, FGD 5)

Most nonusers mentioned that their behaviors related to cannabis have not changed since legalization, as cannabis use is not appealing. However, a few of nonusers mentioned that legalization has removed a barrier to trying cannabis in the future.

I think with this new reality when if I turn 21 and someone like…gives me cocaine or heroin. I would just "No! cause it's … illegal. But since cannabis is legal I might not reject trying it if it was legal for me to do so.

(Taylor, nonuser, FGD 6)

### Seeking safety when purchasing cannabis

This theme illustrates how participants understood and discussed their concern of “safety” in relation to different purchasing alternatives. Whether from dispensaries or dealers, cannabis users stressed the importance of safety and purchasing from “trustworthy” sources. Participants framed “trustworthy sources” as people they knew, including family members and friends.

I always had friends that I trusted enough that they wouldn't try to slip anything or mix with anything. So, it always has been like trusting the people you get it from.

(Olivia, regular user, FGD5)

Other participants under 21 years old described using social media, such as Snapchat, to purchase cannabis. Participants described Snapchat as a private and convenient way to purchase cannabis.

I mean if you're younger…definitely safer to Snap…with your parents…definitely safer to Snapchat someone. They don't know what you said…they don't go all the way vs. your text messages or ongoing talking codes or something…

(Sarah, regular user, FGD8)

Participants related safety not only to the trustworthy sources but also to the ability to recognize the cannabis products’ quality. Most of the participants related the quality of cannabis products to not being mixed with other, unwanted substances.

I feel that I can definitely recognize if someone laced my weed if I didn't know what I was smoking.

(Ben, regular user, FGD 8)

Despite users’ confidence in their “trustworthy” sources, most users and nonusers perceived the dispensary as the safest and the most reliable place to purchase cannabis products.

I think I just want…in my mind, it [a dispensary product] is safer…and I know what kind it is. I know exactly where I am getting it from versus like so-and-so in the back yard "here you go.

(Sarah, regular user, FGD8)

These perceptions were grounded in different explanations. Participants described how the dispensary was regulated by the State, how it offers legitimate products, and how cannabis packaging was a reliable source of information about the products’ components.

Definitely dispensary…It's much nicer to be able to choose what you want and have it like controlled and regulated…I think.

(Sally, occasional user, FGD 7)

(…) cause like from a previous experience, my friend has bought some edibles from there [the dispensary] …and they're just… really strong and legit…I would believe the dispensary.

(Dom, occasional user, FGD 4)

In addition to safety, participants mentioned another advantage of using the dispensary compared to other purchase methods: accessing a variety of products and being able to choose. Several participants mentioned that dealers’ variety of products is limited, but dispensaries were reliable sources of a wide range of products.

So, I don't know…just like it's hard because you get to pick and choose…and you can be picky at the club [the dispensary]. But, you go to the street and you're like ‘I don't get wide variety, but I get cheap and good stuff.

(Chole, regular user, FGD5)

However, some participants found that high cost and the age restriction were the main facilitators to continued use of the black market to purchase cannabis in Nevada. While a few users under 21 years old described purchasing dispensary products through friends or family who could legally access the dispensary, none personally accessed a dispensary. One participant described using a dispensary to purchase cannabis, but she had legal access due to a medical cannabis card.

Since none of us can buy it from the dispensary we just like obviously buy it from dealers…but, I mean most of the time I would never buy it from a dispensary if I have the choice…just because you can get it for so much cheaper….

(José, occasional user, FGD4)

Participants related the high costs of the dispensary products to taxation. Some users mentioned that their purchase habits were primarily driven by cannabis prices.

My opinion… the dispensary was…it was like very systematic…very friendly. But, they were only able to be paid with cash…. kinda throw me off… the prices…were definitely very higher…probably for like tax reasons.

(Soran, occasional user, FGD7)

## Discussion

The overall aim of this study was to understand young adults’ cannabis-related attitudes, perceptions, and behaviors in a state where recreational cannabis use was legal. We found that despite their limited knowledge of Nevada’s cannabis regulations, the majority of participants understood and supported recreational cannabis use legalization using a harm reduction perspective. As in previous studies, increased tax revenue, decreased crime associated with an illegal drug market, and improved product safety were participants’ main arguments for supporting legalization []. Similarly, our participants believed that recreational cannabis legalization has opened a legally, socially, and behaviorally “safer” alternative to purchase cannabis as compared to the informal black market.

In line with previous quantitative studies [8–10], most of our participants did not believe that cannabis legalization affected their cannabis use or non-use. However, participants associated recreational legalization with increased de-stigmatization and acceptability of cannabis use, especially from members of their family, which may affect their future use. This finding is consistent with a previous study conducted among adolescents in Colorado [27] where participants related recreational legalization to increased normalization of cannabis use. Future studies should explore how recreational cannabis use legalization affects young adults’ cannabis use, mediated by perceived acceptability of cannabis use in this age group.

Legalization was also related to access to “safer” cannabis products. Participants perceived the dispensary products as high quality and reliable, primarily because the dispensary is regulated by the state government. Despite these positive perceptions, most users preferred to use the black market. Outside of the mandated minimum legal age limit, high cost, understood as a consequence of taxation, was the main barrier to using the dispensary. In this sense, taxation was perceived as not only a beneficial measure for the community, but also a driver to use the untaxed black market. Price is a well-known determinant of black market use [28, 29], but lower price also facilitates greater use on a population level, which could be detrimental to public health. Although participants indicated that their preference for black market cannabis products is primarily driven by price, they also noted easy access, confidence in identifying high quality “street products,” and trust of their “street sellers” [30] as additional reasons to use the informal black market. Our findings suggest that Nevada’s current legal recreational cannabis infrastructure does not encourage price-sensitive young adult cannabis users of legal purchase age to abandon the high-risk black market for the regulated, legal cannabis market.

### Strengths and limitations

As with all focus group studies, the degree of transferrability of our findings to other contexts is debatable. We have provided a detailed description of the study context to allow readers to evaluate the transferability of our findings. Despite this, our focus group study also has two notable strengths. First, we took several measures to enhance the study’s dependability and the confirmability [31], including creation of field notes during the focus groups and use of quotations to illustrate themes. Second, to enhance the study’s credibility, different researchers with different backgrounds and training were involved throughout the research process, including the data analyses.

## Conclusions

Although nearly all participants perceived that recreational cannabis legalization had not affected their cannabis use behaviors, they understood legalization as creating a legally, socially, and behaviorally “safer” environment to purchase and use cannabis, both at the dispensary and in the black market. Our findings suggest that further investigation of the perceived normalization of cannabis use, in addition to the typical perceived harm and availability, may help explain cannabis use initiation or failure to use the legal market.

## Data Availability

The data that support the findings of this study are available from the corresponding author, NA, upon reasonable request.

## Abbreviations

UNR: University of Nevada, Reno
FGD: Focus group discussion
